# Supplementary material for: Expression of Functional Cannabinoid Type-1 (CB1) Receptor in Mitochondria of White Adipocytes
Source: Cells. 2022 Aug 19;11(16):2582. doi: 10.3390/cells11162582 (PMC9406404; doi:10.3390/cells11162582)
Supplement: Supplementary file 1 [file cells-11-02582-s001.zip › cells-1809455-supplementary.pdf]

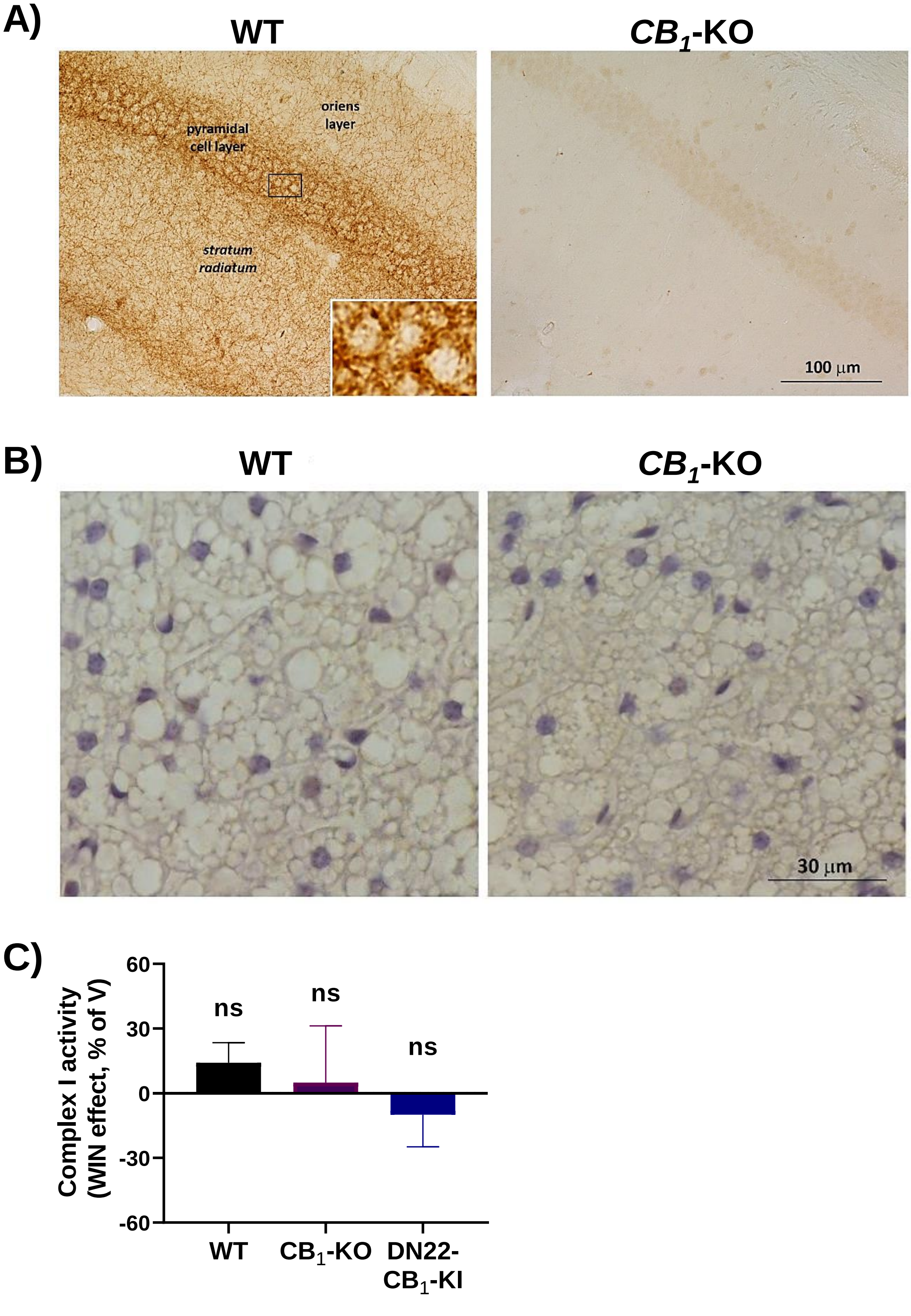

Figure S1

A) Immunohistochemical detection of CB_1 receptor in CA1 hippocampal region of WT vs *CB_1*-KO mice.

B) Representative micrograph of CB_1 receptor immunoreactivity in iBAT of WT vs *CB_1*-KO mice.

C) WIN55.212 (WIN 5µM) effect (relative to vehicle, V) on complex I activity in iBAT from WT, *CB_1*-KO and DN22-*CB_1*-KI mice.

Figure	Conditions	"n" (x group)	Analysis (post-hoc reported in figure)	Factor analyzed	F Ratios	P value
**1C**	(WT vs *CB1*-KO) Total particles	6	Mann-Whitney test			P < 0.0001
**1D**	(WT vs *CB1*-KO) PM particles	6	Mann-Whitney test			P = 0.0022
**1E**	(WT vs *CB1*-KO) Nuclear particles	6	Mann-Whitney test			P = 0.0260
**1F**	(WT vs *CB1*-KO) Cytoplasm particles	6	Mann-Whitney test			P = 0.1797
**1G**	(WT vs *CB1*-KO) MT particles	6	Mann-Whitney test			P = 0.0022
**1H**	(WT vs *CB1*-KO) MT "external" particles	6	Mann-Whitney test			P = 0.002
**1H**	(WT vs *CB1*-KO) MT "inside" particles	6	Mann-Whitney test			P = 0.0022
**1I**	(WT vs *CB1*-KO) Mitochondria	6	Mann-Whitney test			P =0.8182
**1J**	(WT vs *CB1*-KO) CB1+ Mitochondria	6	Mann-Whitney test			P = 0.0022
	WT WIN vs veh	4	One sample t-test			P = 0.028
**2A**	*CB1*-KO WIN vs veh	4	One sample t-test			P = 0.8794
	DN22-*CB1*-KI WIN vs veh	4	One sample t-test			P = 0.6645
	WT, *CB1*-KO, DN22-*CB1*-KI (WIN effect)	4	One way ANOVA (Kruskal-Wallis)		F (3, 12) = 6.146	P = 0.0346
	WT WIN vs veh	4	One sample t-test			P = 0.2279
**S1C**	*CB1*-KO WIN vs veh	4	One sample t-test			P = 0.8645
	DN22-*CB1*-KI WIN vs veh	4	One sample t-test			P = 0.5522
	WT, *CB1*-KO, DN22-*CB1*-KI (WIN effect)	4	One way ANOVA (Kruskal-Wallis)		F (3, 12) = 1.077	P = 0.6298
					Interaction F (3, 36) = 52.33	P < 0.0001
**2C**	Veh - WIN / WT OXPHOS	7	Two way ANOVA (Tukey)	Treatment x Time	Time F (1, 12) = 52.26	P < 0.0001
					Treatment F (1, 12) = 63.18	P < 0.0001
					Interaction F (3, 18) = 2.966	P = 0.0597
**2D**	Veh - WIN / *CB1*-KO OXPHOS	4	Two way ANOVA (Tukey)	Treatment x Time	Time F (1, 9) = 9.441	P = 0.0086
					Treatment F (1, 12) = 3.561	P = 0.1081
					Interaction F (3, 18) = 0.1173	P = 0.9488
**2E**	Veh - WIN / Ati-*CB1*-KO OXPHOS	4	Two way ANOVA (Tukey)	Treatment x Time	Time F (1, 6) = 15.64	P < 0.0001
					Treatment F (1, 6) = 0.1072	P = 0.7545
					Interaction F (3, 18) = 0.2201	P = 0.8811
**2F**	Veh - WIN / DN22-*CB1*-KI OXPHOS	4	Two way ANOVA (Tukey)	Treatment x Time	Time F (1, 6) = 15.64	P = 0.0827
					Treatment F (1, 6) = 0.1651	P = 0.6986
	WT WIN vs veh	7	One sample t-test			P < 0.0001
	CB1-KO WIN vs veh	4	One sample t-test			P = 0.2320
**2G**	Ati-*CB1*-KO WIN vs veh	4	One sample t-test			P = 0.7055
	DN22-*CB1*-KI WIN vs veh	4	One sample t-test			P = 0.6787
	WT, *CB1*-KO, DN22-*CB1*-KI (WIN effect)	4-7	One way ANOVA (Kruskal-Wallis)		F (4, 19) = 12.99	P = 0.0003
	WIN 0.1µM vs veh	7	One sample t-test			P = 0.5970
**3D**	WIN 1 µM vs veh	5	One sample t-test			P = 0.092
	WIN 2 µM vs veh	5	One sample t-test			P = 0.0044
	WIN1µM vs veh / veh TREAT	17	One sample t-test			P = 0.0004
	WIN1µM vs veh / veh JD TREAT	11	One sample t-test			P = 0.8392
	WIN1µM vs veh / veh KH7 TREAT	8	One sample t-test			P = 0.5416
**3E**	WIN1µM vs veh / veh 8-br-cAMP TREAT	6	One sample t-test			P = 0.6591
	veh TREAT vs JD TREAT	17-11	Mann Whitney test			P = 0.0263
	veh TREAT vs KH7 TREAT	17-8	Mann Whitney test			P = 0.0376
	veh TREAT vs 8-br-cAMP TREAT	17-6	Mann Whitney test			P = 0.0171
	WIN1µM vs veh / veh pre TREAT	17	One sample t-test			P = 0.0006
	WIN1µM vs veh / veh JD TREAT	11	One sample t-test			P = 0.3654
	WIN1µM vs veh / veh KH7 TREAT	8	One sample t-test			P = 0.2650
**3F**	WIN1µM vs veh / veh 8-br-cAMP TREAT	6	One sample t-test			P = 0.8791
	veh TREAT vs JD TREAT	17-11	Mann Whitney test			P = 0.0433
	veh TREAT vs KH7 TREAT	17-8	Mann Whitney test			P = 0.0304
	veh TREAT vs 8-br-cAMP TREAT	17-6	Mann Whitney test			P = 0.0133

Figure S2

Statistical analyses related to figures 1-3 and S1C
